# Oxygen Sensing in *Drosophila*: Multiple Isoforms of the Prolyl Hydroxylase Fatiga Have Different Capacity to Regulate HIFα/Sima

**DOI:** 10.1371/journal.pone.0012390

**Published:** 2010-08-25

**Authors:** Julieta M. Acevedo, Lazaro Centanin, Andrés Dekanty, Pablo Wappner

**Affiliations:** 1 Instituto Leloir, Buenos Aires, Argentina; 2 Facultad de Ciencias Exactas y Naturales (FCEyN), Universidad de Buenos Aires, Buenos Aires, Argentina; 3 Consejo Nacional de Investigaciones Científicas y Técnicas, Buenos Aires, Argentina; National Cancer Institute, United States of America

## Abstract

**Background:**

The Hypoxia Inducible Factor (HIF) mediates cellular adaptations to low oxygen. Prolyl-4-hydroxylases are oxygen sensors that hydroxylate the HIF alpha-subunit, promoting its proteasomal degradation in normoxia. Three HIF-prolyl hydroxylases, encoded by independent genes, PHD1, PHD2, and PHD3, occur in mammals. PHD2, the longest PHD isoform includes a MYND domain, whose biochemical function is unclear. PHD2 and PHD3 genes are induced in hypoxia to shut down HIF dependent transcription upon reoxygenation, while expression of PHD1 is oxygen-independent. The physiologic significance of the diversity of the PHD oxygen sensors is intriguing.

**Methodology and Principal Findings:**

We have analyzed the *Drosophila* PHD locus, fatiga, which encodes 3 isoforms, FgaA, FgaB and FgaC that are originated through a combination of alternative initiation of transcription and alternative splicing. FgaA includes a MYND domain and is homologous to PHD2, while FgaB and FgaC are shorter isoforms most similar to PHD3. Through a combination of genetic experiments in vivo and molecular analyses in cell culture, we show that *fgaB* but not *fgaA* is induced in hypoxia, in a Sima-dependent manner, through a HIF-Responsive Element localized in the first intron of *fgaA*. The regulatory capacity of FgaB is stronger than that of FgaA, as complete reversion of *fga* loss-of-function phenotypes is observed upon transgenic expression of the former, and only partial rescue occurs after expression of the latter.

**Conclusions and Significance:**

Diversity of PHD isoforms is a conserved feature in evolution. As in mammals, there are hypoxia-inducible and non-inducible *Drosophila* PHDs, and a fly isoform including a MYND domain co-exists with isoforms lacking this domain. Our results suggest that the isoform devoid of a MYND domain has stronger regulatory capacity than that including this domain.

## Introduction

In response to oxygen deprivation (hypoxia) cells, tissues and whole organisms induce the expression of a wide range of genes that tend to restore energy homeostasis. Hypoxic gene induction is mainly mediated by the Hypoxia Inducible Factor (HIF), a heterodimeric α/β transcription factor composed of two basic-Helix-Loop-Helix-PAS (bHLH-PAS) subunits [1]. Whereas the HIFβ subunit is constitutive, HIFα is tightly regulated by oxygen levels through various mechanisms that include protein stability, transcription coactivator recruitment and subcellular localization [2]–[4]. The molecular mechanism that controls HIFα protein stability has been characterized in detail: In normoxia, HIFα is ubiquitinated and degraded at the 26S proteasome, while in hypoxia the protein is stabilized. HIFα ubiquitination in nomoxia is mediated by the Von Hippel Lindau (VHL) tumor suppressor factor which is the substrate recognition subunit of a multimeric E3 ubiquitin ligase complex [5], [6]. Physical interaction between VHL and HIFα requires hydroxylation of 2 key prolyl residues in the HIFα sequence (P402 and P564 in human HIF-1α), which is catalyzed by specific prolyl-4-hydroxylases, named PHD1- PHD2 and PHD3 [7], [8]. These enzymes are members of the Fe (II) and 2-oxoglutarate dependent dioxygenase superfamily that utilizes O_2_ as a co-substrate for catalysis [6]–[8]. Under hypoxia, PHD hydroxylase activity is reduced, HIFα escapes hydroxylation and proteolysis, leading to HIF nuclear accumulation and transcriptional induction of target genes [7]–[9]. HIF-dependent transcription involves direct binding to Hypoxia Response Elements (HREs) that are characterized by an invariant 5′CGTG 3′ core consensus [10]–[12]. Interestingly, a negative feed back loop, limiting HIFα activity in chronic hypoxia or upon re-oxygenation has been reported: PHD2 and PHD3 mRNAs are induced by low oxygen in a HIF-dependent manner to shut-down HIF activity; PHD1 transcription is oxygen-independent [8], [13].

The occurrence of three mammalian PHD isoforms encoded by three independent genes (PHD 1, PHD2 and PHD3) has opened the question of how each of these enzymes contributes to HIF regulation. It has been shown that all three PHDs can hydroxylate HIFα in vitro, and that upon over-expression, they can all suppress HRE-reporter induction [7]–[8], [14]. Cell culture analysis revealed that, PHD2 has a dominant role in controlling HIF-1α in normoxia [15], while PHD3 is important for regulating HIF in hypoxia or upon re-oxygenation [13]. Furthermore, in vivo studies showed that PHD2, but not PHD1 or PHD3 knockout mice, exhibit enhanced angiogenesis and erythropoiesis [16], [17], whereas PHD1 knockout mice display metabolic differences under ischemic conditions [18].

Previous work from our and other laboratories has led to the identification of a hypoxia response system in the fruit fly *Drosophila melanogaster* that is homologous to mammalian HIF, in which the bHLH-PAS protein Similar (Sima), and the prolyl-4-hydroxylase Fatiga (Fga) are the homologues of HIFα and PHDs, respectively [19]. *sima* null mutant individuals are unable to carry out transcriptional responses to hypoxia, although they are fully viable in normoxia. *fga* loss-of-function alleles showed different levels of Sima accumulation in normoxia, as well as tracheal defects and lethality at different developmental stages. Interestingly, *sima* loss-of-function mutations rescued viability and tracheal defects of *fatiga* mutants, suggesting that Sima protein over-accumulation accounts for these phenotypes [20].

In this work we have performed a characterization of the single *fatiga* locus. We show that the locus encodes three Fatiga variants, FgaA, FgaB and FgaC that originate from a combination of alternative transcription initiation and alternative mRNA splicing. FgaA includes a MYND domain, so it is homologous to PHD2, while both FgaB and FgaC are shorter isoforms that lack the MYND domain, and are similar to PHD3. We have analyzed the expression pattern of FgaA and FgaB, as well as their transcriptional induction in hypoxia. Whereas FgaA expression remains constant and relatively low throughout the life cycle, FgaB is strongly upregulated in adult stages. FgaB but not FgaA is induced in hypoxia in a Sima dependent manner, both in cell culture and in vivo. Cell culture studies revealed that an HRE lying 759 to 756 base pairs upstream of the FgaB transcription initiation site accounts for FgaB induction in hypoxia. Finally, we explored the ability of FgaA and FgaB to shut down Sima-dependent gene expression, finding that, although the two isoforms are active, the regulatory capacity of FgaB is clearly stronger than that of FgaA.

## Results

### The *fatiga* locus: Three PHD isoforms encoded by one single gene

**Figure 1:**
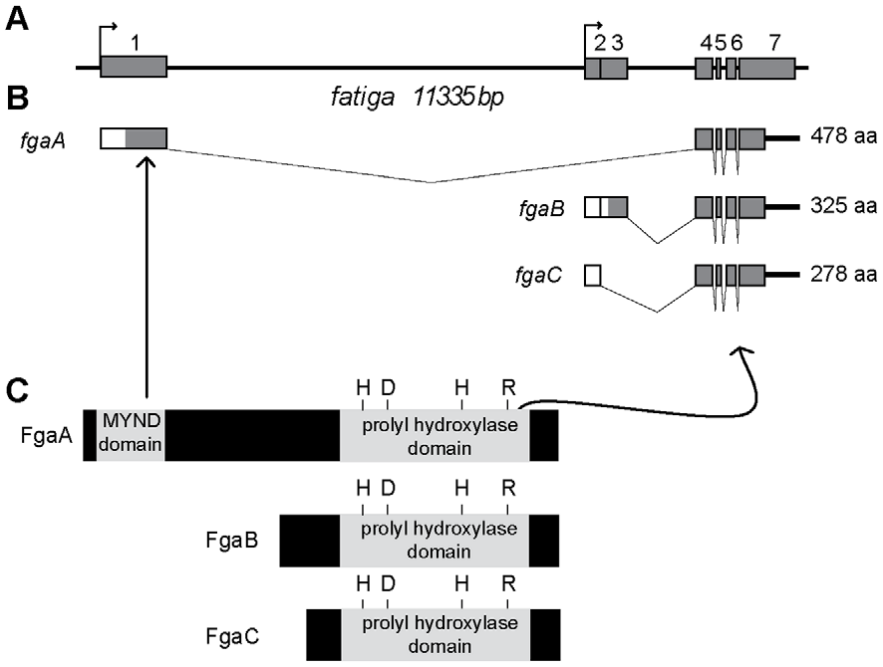
The *fatiga* gene locus. A) Schematic representation of the *fga* locus. Grey boxes represent exons, black lines are introns and arrows indicate transcription initiation sites. B) *fgaA*, *fgaB* and *fgaC* transcripts are generated by a combination of alternative splicing and alternative initiation of transcription. Coding regions are represented by grey boxes, and untranslated regions (UTRs) are representated by white boxes. C) All three transcripts give rise to proteins containing a prolyl hydroxylase domain, whereas only FgaA has a MYND domain.

To study the *fga* locus, we began by seeking for Expressed Sequence Tags (ESTs) in various available cDNA libraries. *In silico* analysis of the locus, and sequencing of 8 representative ESTs revealed that the gene encompasses 7 exons and 5 introns (Fig. 1A), and encodes three different transcripts, which we have named, *fatigaA (fgaA)*, *fatigaB (fgaB)* and *fatigaC (fgaC)* (Fig. 1B). The three transcripts are apparently generated by a combination of alternative transcription initiation and alternative splicing (Fig. 1B). *fgaB and fgaC* share the same transcription initiation site which is different from that of *fgaA*. All three transcripts share an identical 3′region (exons 4 to 7) that encodes the prolyl-hydroxylase domain, but differ in the 5′region. *fgaA* has an exclusive exon (exon 1) that encodes 186 amino acids including a cystein-rich zinc finger domain, called MYND domain, similar to that of mammalian PHD2 (Fig. 1C and Fig. S1). *fgaB and fgaC* are identical except for a small 5′ exon which is exclusive of *fgaB* (exon 3); both FgaB and FgaC are highly similar to PHD3 (Fig. S2). Mammalian PHD1, which displays a unique N terminal stretch of amino acids (168 aa in the human enzyme) has no obvious homologue in the fruit fly (Fig. S2). As *fgaB and fgaC* are almost identical, and share their regulatory regions, we sought to compare *fgaA* and *fgaB* expression patterns, and to explore whether functional differences occur between these two isoforms.

### *fgaB* but not *fgaA* is induced in hypoxia in a Sima-dependent manner

**Figure 2:**
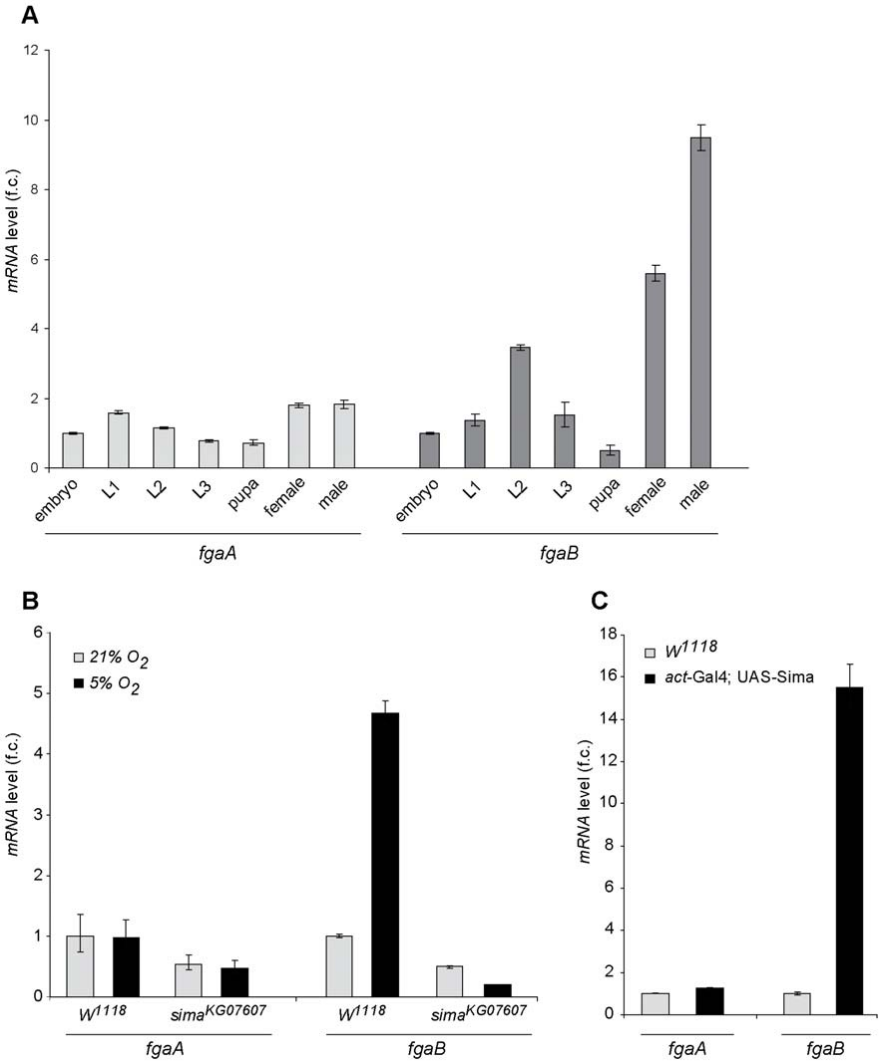
*fgaB* but not *fgaA* is induced in hypoxia in a Sima-dependent manner. A) Relative expression of *fgaA* and *fgaB* during development, as measured by real time PCR, and shown as fold-change expression. Whereas expression levels of *fgaA* remain constant, expression of the *fgaB* transcript rises at the adult stage. Error bars represent standard deviations. B) *fgaB* but not *fgaA* is induced in hypoxia (5% O_2_). In *sima^07607^* homozygous mutant embryos this induction is completely abrogated. Fold-change expression levels are relative to the expression levels in wild type (*w^1118^*) embryos maintained in normoxia; error bars represent standard deviations. C) *fgaB* transcript levels are strongly upregulated in embryos over-expressing Sima. *fgaA* and *fgaB* mRNA expression as assessed by real time PCR; fold changes are relative to *w^1118^* expression levels.

We began by investigating if differences between *fgaA* and *fgaB* expression patterns occur. Real time- PCR analysis revealed that the two transcripts are expressed throughout the *Drosophila* life cycle (Fig. 2A), although *fgaB* but not *fgaA* was strongly upregulated in the adult stage. As mammalian PHD mRNAs are differentially induced by hypoxia, we next analyzed how the different *fga* isoforms respond to oxygen deprivation. We found that *fgaB* but not *fgaA* mRNA is induced in fly embryos exposed to hypoxia (5% O_2_), and that this induction is Sima-dependent, as it was completely blocked in *sima* mutant embryos (Fig. 2B). Consistent with this, ubiquitous over-expression of Sima in transgenic embryos led to *fgaB* but not *fgaA* mRNA upregulation (Fig. 2C). These results indicate that *fgaB* expression is induced in hypoxia in a Sima/HIF dependent manner, while *fgaA* mRNA transcription is oxygen-independent.

### The *fgaB* regulatory region contains a functional HIF Responsive Element

**Figure 3:**
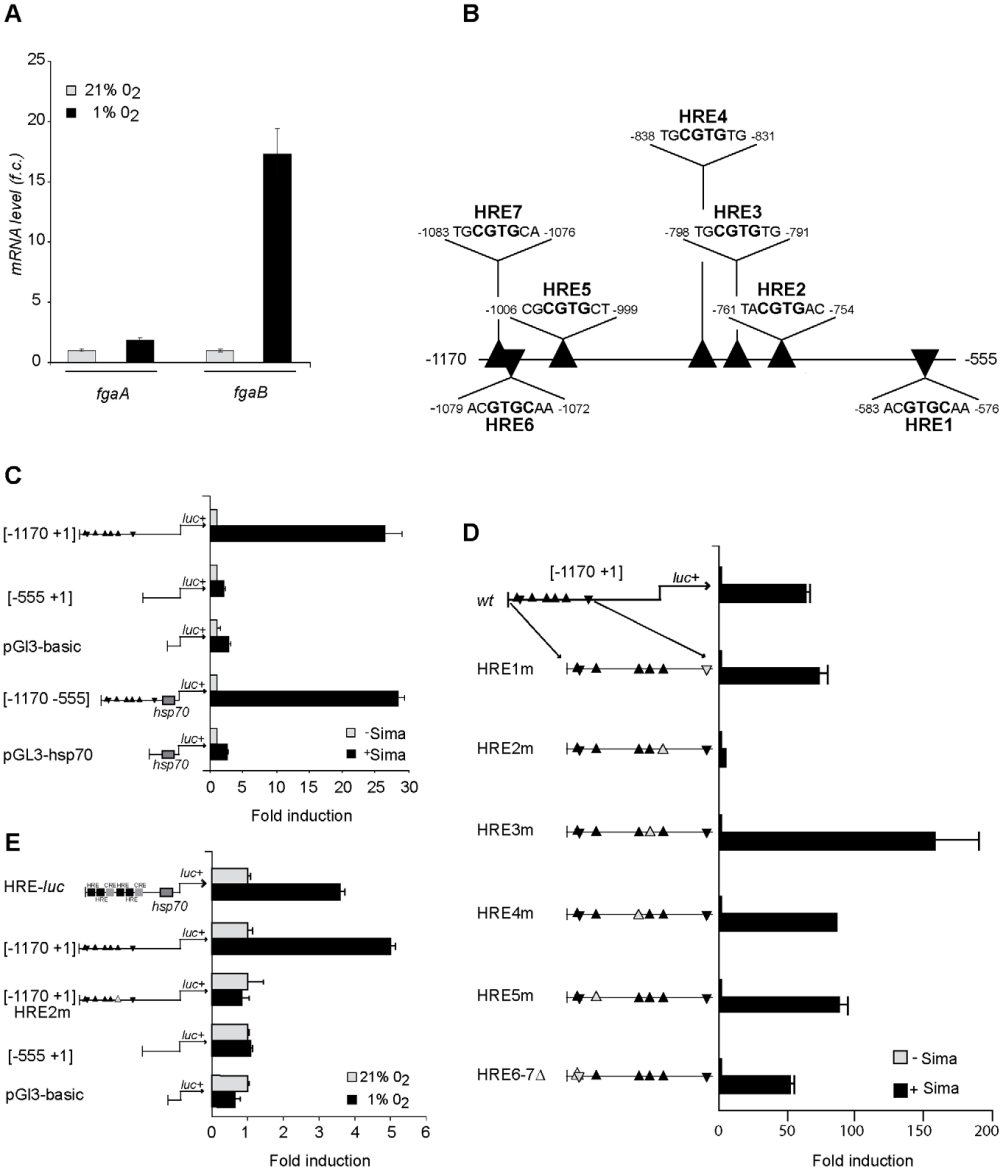
The *fgaB* regulatory region contains a functional HIF Responsive Element. A) *fgaB* but not *fgaA* mRNA is induced in S2 cells exposed to hypoxia (1% O_2_). Real time-PCR analysis; fold-change expression levels are relative to expression in normoxia; error bars represent standard deviations. B) Schematic representation of the *fgaB* [−1170 −555] regulatory region. The black triangles represent putative HIF Responsive Elements (HREs). The sequence and position of presumptive HREs 1 to 7 is indicated; the HRE core consensus is highlighted in bold font. C) The [−1170 −555] DNA fragment includes functional HRE sequences. The [−1170 +1], [−555 +1] and [−1170 −555] luciferase reporter constructs were transiently co trasfected in S_2_ cells, along with the Sima expression vector pAc5.1-Sima (+Sima) or along with empty vector pAc5.1-V5 (-Sima). pGl3-basic or pGl3-Hsp vectors were included as controls. Whereas the [−555 +1] fragment fails to induce Sima-dependent transcription of the reporter, the fragments [−1170 +1] and [−1170 −555] provoke strong induction of the reporter. Luciferase activity is expressed as fold induction relative to that of the corresponding empty control vector. Error bars represent the standard deviation of duplicate luciferase determinations. Each experiment was repeated at least 3 times; one representative experiment is shown. D) The only functional HRE in the [−1170 +1] interval is HRE2. S_2_ cells were transiently transfected with the reporter construct [−1170 +1] containing all 7 wild type HREs or each of the mutagenized versions of the [−1170 +1] reporter (HRE1m to HRE5m and HRE6-7Δ). Each of these reporters was co-transfected along with a Sima expression plasmid (+Sima) or with an empty vector (-Sima). Expression of Sima upregulated all the reporters with the exception of HRE2m. Data are shown as in A and B. (E) HRE-dependent induction in S2 cells exposed to hypoxia: The reporter constucts [−1170 +1], [−555 +1] and [1170 +1] HRE 2m, as well as the pGl3-basic empty vector were transiently transfected in S2 cells, which were later transferred to hypoxia (1%O_2_) or maintained in normoxia (21% O_2_), after which luciferase activity was assessed. *HRE-luc* reporter was included as a hypoxia control. The [−1170 +1] reporter was strongly induced in hypoxia, while the expression of the reporters [−555 +1] and [1170 +1] HRE 2m did not increase in hypoxia. Luciferase activity is presented *as* fold induction relative to that of control cells maintained in normoxia.

Given that in *Drosophila* embryos, *fgaB* hypoxic induction depends on Sima, we next sought to search for candidate hypoxia response elements (HREs) in the *fgaB* regulatory region. These studies were carried out in cultured *Drosophila* S2 cells, which, paralleling the observations carried out in embryos, exhibited hypoxic induction of *fgaB* but not *fgaA* mRNA (Fig. 3A). The *fgaB* 5′ upstream region was found to contain 7 predicted HREs, which were named HRE1 to HRE7 (Fig. 3B). Thus, a DNA fragment including these 7 HREs (*fgaB* [−1170 +1]) was cloned in a luciferase reporter plasmid, and the construct co-transfected in *Drosophila* S2 cells, along with a Sima over-expression vector (pAc5.1- Sima). As shown in figure 3C, over-expression of Sima provoked strong induction of the luciferase reporter, indicating that the [−1170 +1] fragment contains at least one functional HRE. Next, we generated a deletion construct [−555 +1] which does not include any candidate HRE; as expected, no induction of the [−555 +1] luciferase construct was observed, suggesting that the region [−1170 −555] is required for Sima dependent induction of the reporter (Fig. 3C). To confirm this requirement, we generated a new luciferase reporter in which the region [−1170 −555] including all 7 candidate HREs was cloned upstream of an *hsp70* minimal promoter [−1170 −555]. As depicted in figure 3C Sima-dependent induction of this reporter was observed, and induction levels were comparable to those of the full length [−1170 +1] original construct. The next step was to find out which of the 7 presumptive HREs included in the [−1170- +1] interval are responsible for Sima-dependent induction, so we performed point mutations or micro deletions in each of these HREs in the context of the endogenous promoter. In HREs 1 to 5 the core CGTG consensus was mutated to AGTG, whereas HREs 6 and 7 were deleted (Fig. S3). Mutagenesis of HRE2 provoked complete loss of Sima dependent induction of the [−1170 +1] luciferase reporter, while mutation or deletion of any of the other 6 HREs had no effect on reporter induction (Fig. 3D).

Next, we sought to analyze if induction of the luciferase reporters observed upon over-expression of Sima also occurs in cells exposed to hypoxia (1% O_2_). The reporters [−1170 +1], [−555 +1], and the [−1170 +1] including the mutagenized HRE2 were transfected in S2 cells, which were then exposed to hypoxia (1% O_2_). The pattern of luciferase reporter induction in these hypoxic cells was similar to that observed upon over-expression of Sima, although induction in this case was more modest (Fig. 3E). These results confirm that the HRE2 is required for *fgaB* transcriptional induction in hypoxia.

### FatigaA and FatigaB differentially regulate Sima

Given that FgaA and FgaB include different protein domains and have different expression patterns, we sought to address if the two isoforms have different effects on Sima regulation. We have reported before that *fga* loss-of-function mutations leads to accumulation of Sima protein in normoxia, and constitutive expression of a hypoxia-inducible reporter in transgenic lines; abnormal accumulation of Sima in these mutants provokes lethality [20]. Interestingly, before dying, the *fga* mutant larvae exhibit defects in the process of gas filling of the respiratory (tracheal) system, a process that normally occurs by the end of embryogenesis [20].

**Figure 4:**
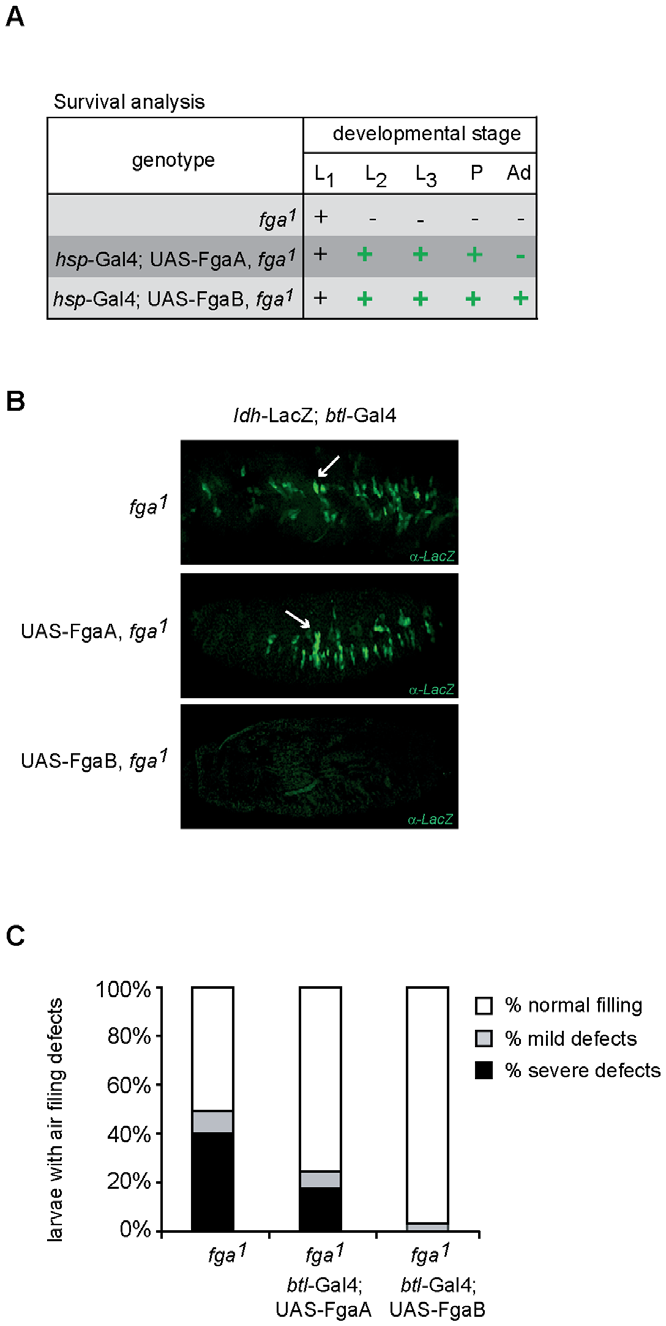
FatigaA and FatigaB differentially regulate Sima. A) Reversion of developmental viability of *fga^1^* homozygous mutants upon transgenic expression of FgaA or FgaB. Expression of FgaB completely reverted the *fga^1^* lethal phenotype, as individuals reached the adult stage. FgaA expression enabled development only to the pupal stage. (+) indicates the occurrence of individuals of the indicated developmental stage; (−) indicates absence of individuals of the mentioned stage. B) Expression of FgaB but not FgaA reverts the constitutive expression of the *ldh*-LacZ reporter that occurs in *fga^1^* mutant embryos in normoxia, as shown in the immunofluorescence micrograph after anti beta-gal staining. The arrow indicates groups of cells that express the *ldh*-LacZ reporter. C) Fga B is more efficient than FgaA in restoring tracheal liquid clearence in *fga^1^* mutant first-instar larvae. Tracheal analysis of *fga^1^* mutant larvae, and of *fga^1^* mutant larvae, expressing either FgaA or FgaB under control of a *btl*-Gal4 driver, was performed under a bright field microscope. Percentage of larvae with air filling defects was quantified, for whose purpose three categories were defined: tracheae full of air (white box), tracheae partially filled with liquid (grey box), and tracheae full of liquid (black box).

Since *fga* loss-of-function mutations compromise the entire *fga* locus, the expression of both FgaA and FgaB is affected in these mutants. Therefore, in order to analyze specific functions of these two Fga isoforms, we sought to restore the expression of FgaA or FgaB in transgenic flies, which are, at the same time, homozygous mutant for *fga^1^*, the strongest available loss-of-function allele of the gene. We initially checked mRNA expression levels of the FgaA and FgaB transgenes were similar (data not shown), so that the biological effect of the two enzymes can be compared. Since *fga^1^* homozygous mutants are lethal at the first larval instar, we analyzed if FgaA or FgaB are capable of rescuing developmental viability of the mutants. Thus, we over-expressed the FgaA or FgaB transgenes in a *fga^1^* mutant background, by using an ubiquitous heat-shock inducible gal4 driver (see materials and methods). FgaB expression fully reverted *fga^1^* lethality, allowing survival to the adult stage, whereas FgaA expression, on the other hand, led to partial rescue of viability, as individuals reached the pupal stage but failed to develop further into viable adults (Fig. 4A).

The above results suggest that FgaB is more efficient than FgaA in rescuing developmental defects provoked by mutation of the *fga* locus, so we next sought to investigate the capacity of each of the two Fga isoforms to suppress normoxic expression of an *ldh*-LacZ HIF-inducible transgenic reporter that occurs in *fga^1^* homozygous mutant embryos. Since this transcriptional reporter is preferentially induced in cells of the tracheal system, we over-expressed FgaA or FgaB in these cells in *fga^1^* homozygous mutant embryos. As depicted in figure 4B, expression of FgaB completely suppressed normoxic expression of the *ldh*-LacZ reporter, whereas FgaA had no effect on the expression of the same reporter.

Finally, we analyzed the ability of the different Fga isoforms to rescue the air filling defects observed in larval tracheal tubules of *fga^1^* mutants. Paralleling the results described above, expression of the FgaA transgenic construct led to partial rescue of tracheal air filling defects of *fga^1^* mutants, upon specific expression of the transgene in the tracheal system. Similar transgenic expression of FgaB reverted the air filling defects almost completely (Fig. 4C), suggesting again that FgaB is more efficient than FgaA in reducing Sima protein levels.

## Discussion

Three PHD variants occur in mammals, and one single PHD gene, named EGL9, has been reported in *Caenorabditis elegans*. In *Drosophila*, previous studies on Fatiga, the *Drosophila* PHD homologous gene, have focused on its role in the regulation of Sima protein abundance [19]–[21] and CyclinD-dependent cellular growth [22]. In these functional studies, however, the occurrence of diverse Fga isoforms has not been addressed. In this work, we have analyzed the *fatiga* locus, revealing that three different PHD isoforms occur in the fruit fly, which are generated through a combination of alternative splicing and alternative initiation of transcription. One of the isoforms, FgaA, includes a MYND domain, so it is homologous to mammalian PHD2, and the other two isoforms, FgaB and FgaC, lack a MYND domain, and are similar to PHD3. Thus, the diversity of PHD isoforms, including or not a MYND domain, seems to be an ancestral condition in evolution maintained in phylogenetically distant phyla such as insects and mammals. The occurrence of a single PHD isoform including a MYND domain in *C. elegans* might be due to evolutionary loss of shorter PHD variants.

In mammals PHD2 and PHD3, but not PHD1 mRNAs, are HIF-inducible [8]. In this work we have shown that FgaB, but not FgaA, is hypoxia-inducible, and that this induction depends on *Drosophila* HIF/Sima. A HIF Responsive Element (HRE) that mediates hypoxic transcriptional activation of *fgaB* mRNAs is localized at the position –759 to –756 with respect to the transcription initiation site of *fgaB*. Most HREs of hypoxia inducible genes of various organisms localize at their 5′ regulatory region no more than 1 Kb upstream to the transcription initiation site [12]. The identified HRE upstream to the *fgaB* open reading frame adjusts to this general rule. Due to the structure of the *fga* locus, the 5′ regulatory region of *fgaB* lies in the large (8630 bp) first intron of *fgaA*.

Sequence conservation of the HRE lying upstream of *Drosophila fgaB* transcription initiation site and the mammalian PHD3 HRE –localized in its first intron- [23] is remarkable, and extends beyond CGTG HRE invariant core. Fourteen out of 17 nucleotides around the *fgaB* HRE (CTGGGCTA**CGTG** AGCAT) are conserved in the PHD3 regulatory region (underlined bases are not conserved). This observation supports the notion that oxygen-dependent induction of PHD isoforms is important for adaptation of organisms to changing oxygen conditions.

The fact that a single *Drosophila* PHD locus encodes different isoforms that parallel two of the mammalian PHD variants encoded by independent genes is remarkable, and argues in favor that a combination of PHDs including or not a MYND domain is functionally relevant. The role of the MYND domain in HIF prolyl-4-hydroxylases is intriguing. Although PHD2 is the most abundant mammalian isoform and hence, has a dominant role in controlling HIFα in normoxia, PHD3 has been reported to have stronger intrinsic hydroxylation capacity than PHD2, which includes the MYND domain [13], [15] . Consistent with this, the MYND domain has been proposed to mediate inhibition of PHD2 hydroxylase activity, as deletion of this domain led to increased activity of the enzyme [24]. Supporting the notion of the MYND domain provoking reduction of PHD regulatory capacity, it has been shown that direct interaction of the peptidyl cis/trans isomerase FKBP38 with the MYND domain of PHD2 negatively regulates PHD2 protein stability [25]. FKBP38 does not interact with the hydroxylase isoforms PHD1 or PHD3, which lack the MYND domain [26] . Some reports, however, weigh in favor of a model of a MYND domain enhancing PHD negative regulation of HIF, as PHD2 but not PHD1 or PHD3 have the capacity to inhibit HIF transcriptional activity through a hydroxylation-independent mechanism [27], [28]. Consistent with this, proteins including a MYND domain have been reported to mediate transcriptional inhibition of other transcription factors [29], so it is conceivable that transcription inhibitory capacity is a general feature of this domain. Thus, it is still unclear as whether the MYND domain increases or decreases the regulatory capacity of PHDs. Our results in *Drosophila* support the latter possibility, as the PHD isoform that lacks the MYND domain has stronger regulatory capacity than the isoform that includes this domain. Detailed biochemical and functional studies are required to define the precise role of this protein domain in transcriptional responses to hypoxia.

## Materials and Methods

### Fly stocks

Flies used in this study were, w^1118^, *en-*Gal4, *act-*Gal4, *btl-*Gal4 and *hsp-*Gal4 (Bloomington *Drosophila* stock center); UAS- Sima and *ldh*-LacZ [19] and *fga^1^* strains have been previously described [20]. Flies were grown in standard culture media at 25°C. Embryos were collected in egg laying agar plates and grown at 25 or 18°C until the desired stage. When necessary, embryos were exposed to hypoxia (5% O_2_) for 4 hours using a Forma Scientific 3131 incubator at 25°C.

### Real Time PCR

Total RNA from embryos, larvae, pupae or adults was isolated using the Trizol reagent (Invitrogen). RNA samples (1–1.5 µg) were reverse-transcribed using the superscript III First-strand synthesis system (Invitrogen), using oligo-dT as a primer. The resulting cDNA was used for real-time PCR (Stratagene MX300 sp), using the hot start Platinum Taq DNA polymerase (Invitrogen) and SYBRGreen and ROX (Invitogen) as fluorescent dyes. *fgaA* and *B* specific primers were used, and samples normalized using *rpl 29* and *tub* primers. Three independent biological samples were analyzed in each experiment. One representative experiment is shown.

### DNA constructs

The region [−1170 +1] of the *fgaB* regulatory region was amplified with specific primers including KpnI and XhoI restriction sites on their 5′ ends, and cloned into the PCR4Blunt-TOPO vector (Invitogen) The fragment of interest was then subcloned in a pGL3 basic vector (Promega), using the KpnI and XhoI restriction sites to obtain the *fgaB* [−1170 +1] reporter constuct. To generate the reporter constructs *fgaB* [−1170 −555] and *fgaB* [−555 +1], the *fgaB* [−1170 +1] reporter was digested with KpnI and Pst1, obtaining 2 products, the fragment [−1170 −555] and the reporter devoid of the [−1170 −555] fragment. The Δ[−1170 −555] reporter was then treated with Klenow DNA polymerase (Promega) to generate blunt ends and then relegated to generate *(fgaB* [−555 +1]). The fragment [−1170 −555] was subcloned in a pEGFP-C1 plasmid (Clontech), using PstI and KpnI restriction sites. Thereafter, the insert was removed by cutting with KpnI and XhoI, and subloned into the pGl3-Hsp vector [30], obtaining the Luciferase reporter *fgaB* [−1170 −555]. Point mutations or micro deletions of candidate HREs were generated by overlap extension PCR. The amplicon containing each mutation or micro deletion was replaced in the *fgaB* [−1170 +1] construct, using KpnI and PstI restriction sites. When necessary, HRE-luc [31] reporter was included as a hypoxia control. To generate the UAS-Fga constructs, the *fgaA* or *fgaB* coding sequences were subcloned from EST LD24638 or GH23732 plasmids into a pCaSpeR-UAS vector using BglII and XhoI.

### Cell transfection, β-gal activity determination and Luciferase assay

Schneider-2 line (S2) cells were maintained at 28°C in Schneider *Drosophila* medium (Sigma) supplemented with 10% fetal bovine serum (Gibco), containing 50 units/ml of penicillin and 50 g/ml of streptomycin in 25 or 75 cm^2^ T-flasks (Greiner). Cells were plated in 12 well plates and 24 h later were transiently trasfected with 6.3 µg of DNA, using the Calcium Phosphate Transfection Kit (Invitrogen), according to instructions of the manufacturer. In all the experiments, cells were transfected with the construct to be analyzed, along with the plasmid pAc5.1-LacZ to normalize transfection efficiency. In experiments in which the effect of the expression of Sima was analyzed, cells were also transfected with the pAc5.1-Sima plasmid [31], or with an empty vector (pAc5.1-V5) that served as a negative control. The day after transfection, cells were scraped and plated in duplicate in 24-well plates. Reporter gene expression was assessed 72 h after transfection using the Steady.Glo Luciferase Assay System (Promega), and luciferase activity was normalized against β*-gal* activity, which was measured using 2-Nitrophenyl β-D-galactopyranoside (ONPG). In experiments analyzing induction by hypoxia, cells were transferred to hypoxia 1% O_2_, 20 h prior to luciferese and β- galactosidase activity determination. Hypoxia was applied using Forma Scientific 3131 incubator at 28°C.

### Survival and tracheal phenotype analysis

Survival analysis was carried out in flies over expressing FgaA or FgaB under control of an *hsp*70-Gal4 diver in a *fga^1^* mutant background by applying 30 minute heat-shocks once a day. Flies were allowed to lay eggs on agar plates, embryos collected for 6–8 h, and first-instar larvae were transferred in groups of 50 to vials containing culture media, and let develop. Tracheal phenotype analysis was performed in first instar larvae, expressing FgaA or FgaB under control of a *btl*-Gal4 driver in a *fga^1^* homozygous mutant background. Resulting larvae were ether anesthetized and observed under a bright-field microscope (Olympus BX-60) for tracheal analysis.

### Analysis of *lhd*- lacZ reporter expression

Expression of the *ldh*-lacZ hypoxia-inducible reporter was assessed in embryos expressing FgaA or FgaB transgenes using a *btl* G4 driver in *fga1* homozygous mutant embyos. Expression of the reporter was assessed by immunofluoresence as previously described [19]. Briefly, embryos were bleach-dechorionated and fixed in 3.7% formaldehyde for 20 minutes, blocked for 2 h in PBS containing 1% bovine serum albumin and 0.1% Triton X-100, and then incubated overnight with a primary anti-beta-gal (Cappel) antibody. After washing, embryos were incubated with a secondary antibody for 2 hours in PBS-Triron containing 10% normal goat serum. Observations were carried-out in a Carl Zeiss LSM5 Pascal confocal microscope.

## Supporting Information

Figure S1 FgaA is homologous to human PHD2. Sequence alignment showing the identity between FgaA and human PHD2. Conserved residues corresponding to the MYND domain are shaded in grey. Amino acids critical for Fe2+ association are marked in black. “*” indicates identical residues; “:” indicates conserved substitutions, and “.” means that a semi-conserved substitution occurred.
(0.18 MB TIF)

Figure S2 FgaB and FgaC are similar to PHD3. Sequence alignment of FgaB, FgaC and human PHD1 and PHD3. PHD1 contains a stretch of 168 amino acids at the N-terminus (grey) that is not present in PHD3. No predicted domain or homology to Drosophila proteins was found in this N-terminal region. FgaB and FgaC are therefore most similar to mammalian PHD3. Amino acids critical for Fe2+ association are marked in black“*” indicates identical residues residues; “:” indicates conserved substitutions, and “.” means that semi-conserved substitutions occurred.
(0.21 MB TIF)

Figure S3 Representation of the deletion performed in HREs 6 and 7 to generare the reporter construct [−1170 −555] HRE 6-7D. A) Sequence of the [−1170 −555] wild type reporter construct containing HREs 6 and 7. Arrows indicate the orientation of the HREs. B) Sequence of the [−1170 −555] HRE 6-7D reporter in which the core sequence of the HREs 6 and 7 has been deleted.
(0.16 MB TIF)
