# Supplementary figures and images for: Oxygen Sensing in Drosophila: Multiple Isoforms of the Prolyl Hydroxylase Fatiga Have Different Capacity to Regulate HIFα/Sima
Source: PLoS One. 2010 Aug 25;5(8):e12390. doi: 10.1371/journal.pone.0012390 (PMC2928329; doi:10.1371/journal.pone.0012390)

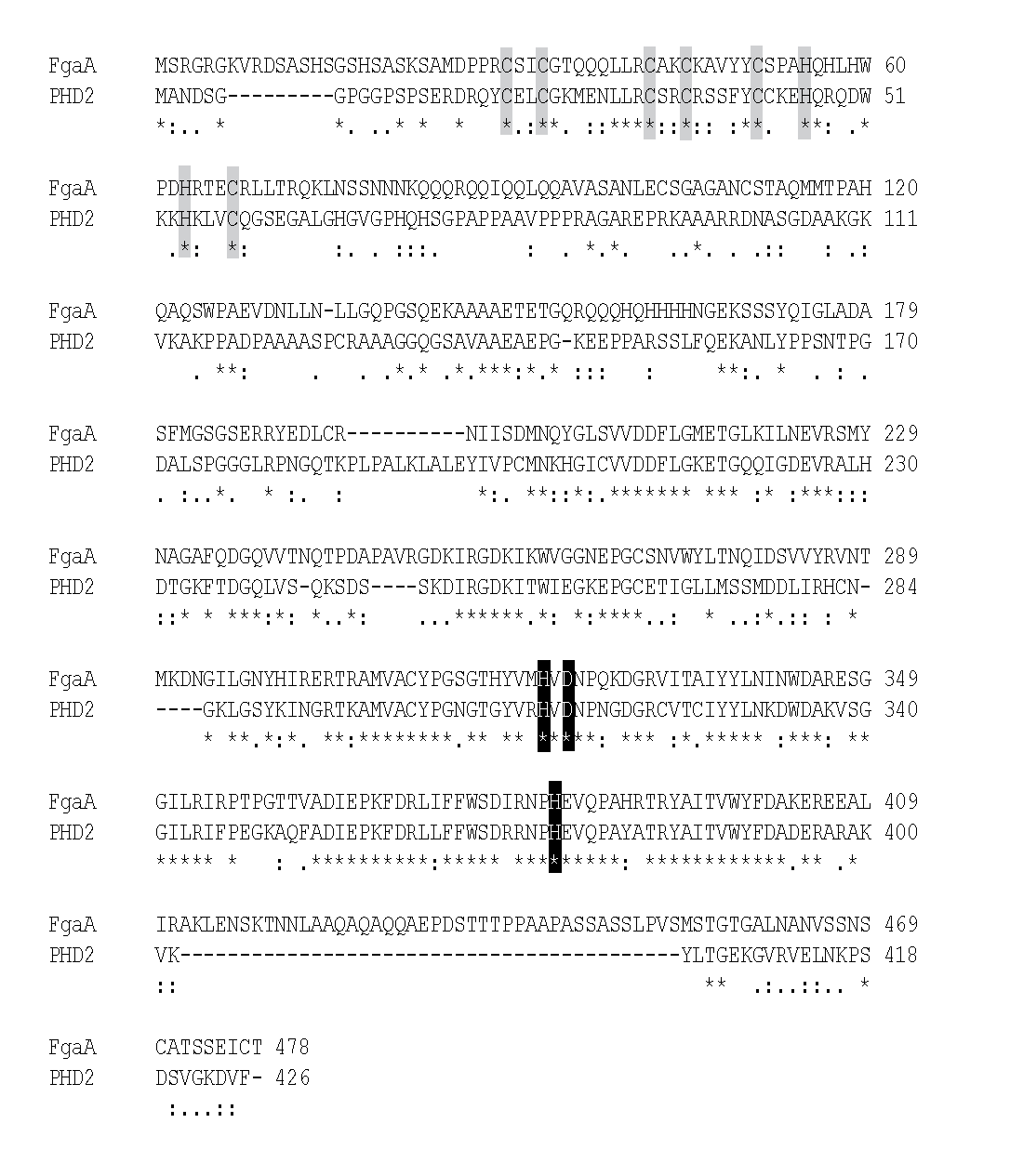

Supplement: Figure S1 — FgaA is homologous to human PHD2. Sequence alignment showing the identity between FgaA and human PHD2. Conserved residues corresponding to the MYND domain are shaded in grey. Amino acids critical for Fe2+ association are marked in black. “*” indicates identical residues; “:” indicates conserved substitutions, and “.” means that a semi-conserved substitution occurred. (0.18 MB TIF) [file pone.0012390.s001.tif]

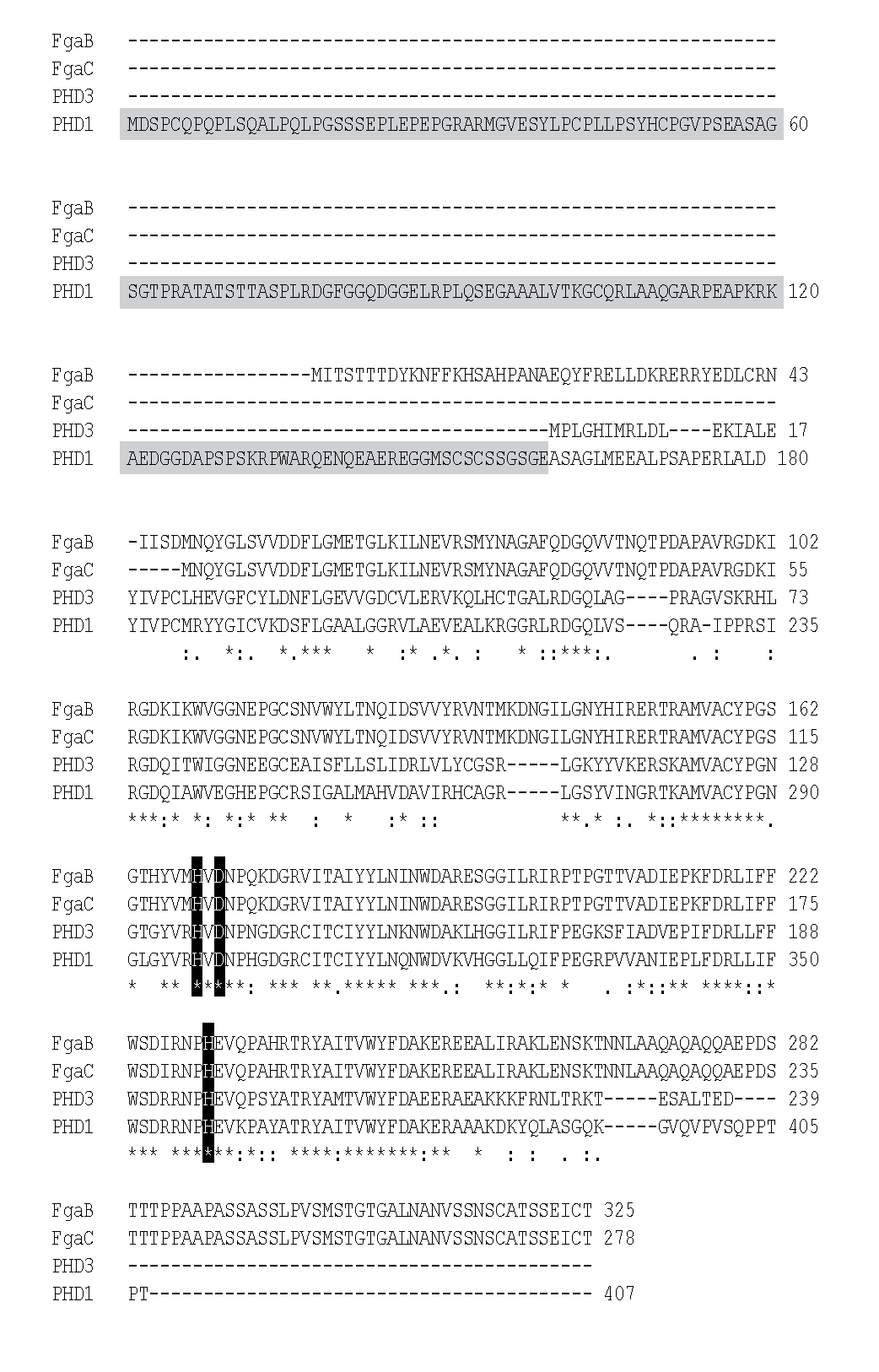

Supplement: Figure S2 — FgaB and FgaC are similar to PHD3. Sequence alignment of FgaB, FgaC and human PHD1 and PHD3. PHD1 contains a stretch of 168 amino acids at the N-terminus (grey) that is not present in PHD3. No predicted domain or homology to Drosophila proteins was found in this N-terminal region. FgaB and FgaC are therefore most similar to mammalian PHD3. Amino acids critical for Fe2+ association are marked in black“*” indicates identical residues residues; “:” indicates conserved substitutions, and “.” means that semi-conserved substitutions occurred. (0.21 MB TIF) [file pone.0012390.s002.tif]

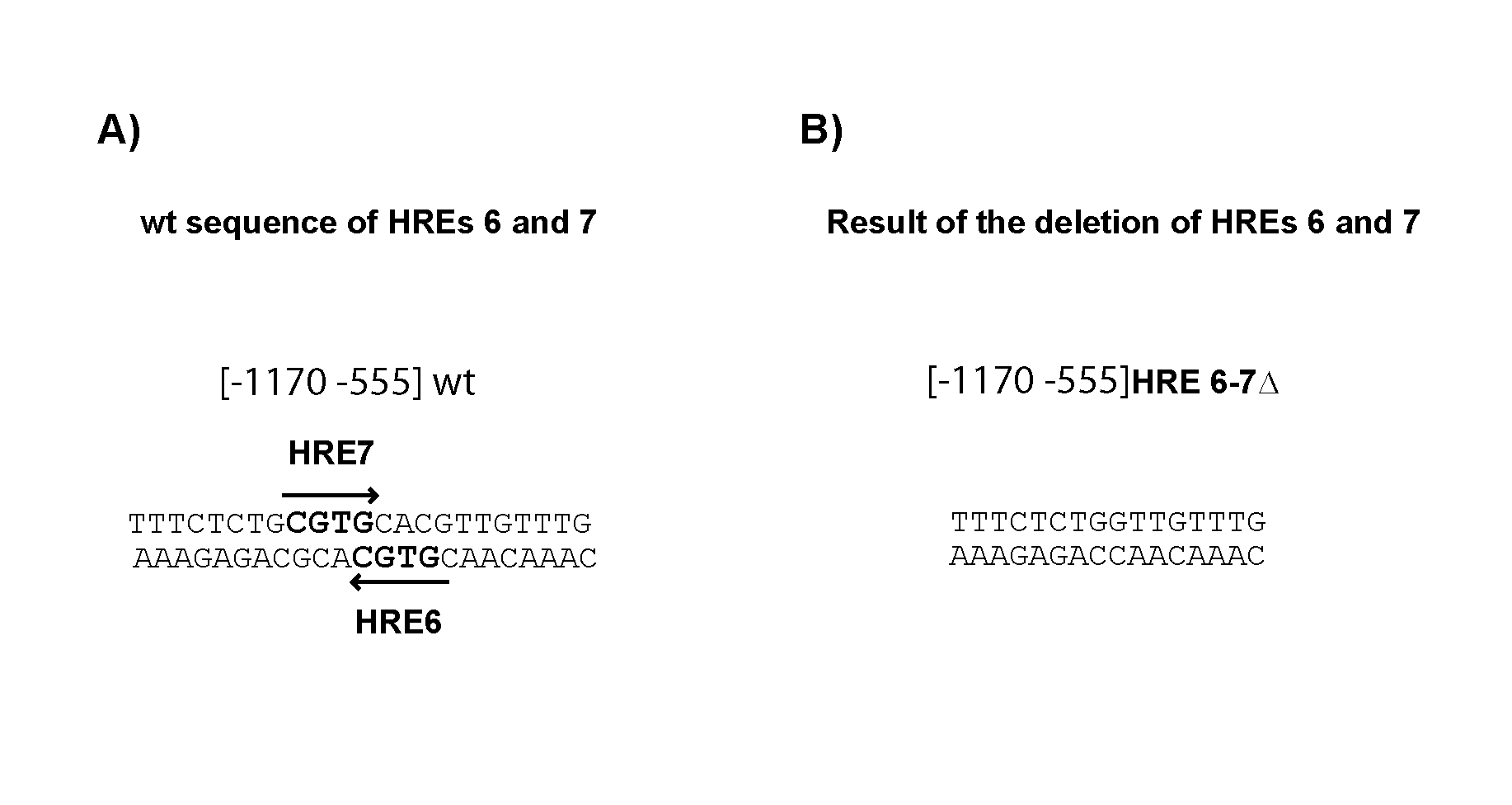

Supplement: Figure S3 — Representation of the deletion performed in HREs 6 and 7 to generare the reporter construct [−1170 −555] HRE 6-7D. A) Sequence of the [−1170 −555] wild type reporter construct containing HREs 6 and 7. Arrows indicate the orientation of the HREs. B) Sequence of the [−1170 −555] HRE 6-7D reporter in which the core sequence of the HREs 6 and 7 has been deleted. (0.16 MB TIF) [file pone.0012390.s003.tif]
